# Predator niche overlap predicts effects on aphid vectors and a vector‐borne virus

**DOI:** 10.1002/eap.70065

**Published:** 2025-07-03

**Authors:** Benjamin W. Lee, Saumik Basu, Liesl Oeller, Tobin D. Northfield, David W. Crowder

**Affiliations:** ^1^ Department of Entomology and Nematology University of California—Davis Davis California USA; ^2^ Department of Entomology Washington State University Pullman Washington USA; ^3^ Tree Fruit Research and Extension Center Washington State University Wenatchee Washington USA

**Keywords:** biodiversity, habitat domain, predation risk, trait‐mediated effects, vector, virus

## Abstract

Multiple predator species can enhance or disrupt prey suppression based on whether different predators forage in complementary or overlapping niches. Interactions between predator species are primarily evaluated by resulting effects on prey abundance, although alterations of prey behavior also occur. When prey are vectors of plant pathogens, changes in their movement among plants may affect pathogen transmission as strongly as changes in vector abundance. Here, we assessed how single predator species, and pairs of species with varying degrees of niche overlap, affected pea aphid vectors and transmission of an aphid‐borne pathogen, pea‐enation mosaic virus (PEMV). Foliar‐foraging predators reduced vector abundance but altered vector behavior in ways that promoted PEMV transmission, resulting in no net effects on PEMV prevalence. Predator pairings also enhanced vector suppression but caused vectors to move to parts of plants that were more susceptible to PEMV. Surprisingly, pathogen prevalence was only reduced in predator pairings that did not exhibit super‐additive predation rates. Our study shows that enhanced predator consumption of vectors due to niche complementarity can affect pathogen transmission differently than it affects vector dispersal and feeding behaviors. Nonetheless, long‐term suppression of vector populations may ultimately reduce pathogen transmission.

## INTRODUCTION

When multiple predator species attack the same prey, interactions between predators range from synergistic to antagonistic, with consequences across trophic levels (Ives et al., 2005; Sih et al., 1998). The predator habitat domain framework defines spatial niches of predators and predicts how multiple predators affect prey (Northfield et al., 2017; Preisser et al., 2007; Schmitz, 2007). Predators that forage in distinct niches may enhance prey suppression by preventing prey from seeking refuges, but predators with overlapping niches may compete or engage in intraguild predation, reducing predation risk (Snyder & Ives, 2001; Straub & Snyder, 2008). Prey behavioral responses to multiple predators can affect population dynamics and trophic cascades if survival, reproduction, or feeding behaviors are affected (Northfield et al., 2010). However, the effects of these behavioral responses on pathogen transmission have rarely been assessed.

Many insect herbivores are vectors of plant viruses (Jones & Naidu, 2019). Plant virus transmission is affected by vector behaviors such as host selection, the duration of feeding, and dispersal (Fereres & Moreno, 2009). Empirical studies and models suggest that even small shifts in these vector behaviors can greatly alter rates of virus spread (Crowder et al., 2019; Eigenbrode et al., 2018). Predators, which consume vectors, can affect virus transmission both by reducing vector abundance and altering vector behaviors (Lee et al., 2021; Long & Finke, 2015). However, such effects on vector abundance and behavior may conflict to limit net effects on virus transmission. A study examining lady beetles' effects on aphid vectors carrying pea‐enation mosaic virus (PEMV) found predators significantly reduced vector abundance but increased vector dispersal between host plants, resulting in no overall effect on virus transmission (Lee et al., 2022). Examining predators' impacts on both vector populations and specific vector behaviors is crucial to understanding their role in virus transmission dynamics.

While theories using habitat domain to predict multiple predator effects on prey abundance are well supported (Woodcock & Heard, 2011), effects on vector behavior and pathogen spread may differ. In a case of predator–predator synergy, a foliar‐foraging lady beetle induced aphid prey to drop from hosts and become vulnerable to a ground beetle (Losey & Denno, 1998a). If aphids were vectors, however, predator‐induced dropping might have accelerated pathogen transmission by increasing vector movement to new plants (Nelson et al., 2004). One study assessing the effects of multiple predators on vector‐borne pathogens found that increasing predator species richness reduced vector abundance but did not influence host plant occupancy by vectors (Long & Finke, 2015). However, predator foraging strategies were not considered, making the importance of interactions between predator species on vectors unclear. Testing habitat domain theories of multiple predators on pathogens will require comparisons between predators that forage across varied habitats alongside measurements of vector behaviors.

Here, we expanded on a habitat domain framework for multiple predator effects to include vector behaviors that mediate pathogen transmission, including feeding location and movement, by assessing how three predator species affected an aphid vector and a viral plant pathogen. We manipulated the composition of predator communities in a substitutive experiment and assessed how the individual and combined effects of foliar and ground‐foraging predators with different levels of niche overlap affected vector abundance, feeding location, dispersal, and virus transmission. We hypothesize that interactions among multiple predators will have variable but predictable effects on vector abundance, movement, and feeding behavior. Expanding the habitat domain framework to consider prey behavior in addition to abundance would enhance our understanding of how multiple predators affect prey and how these effects cascade through ecosystems.

## METHODS

### Study system

The Palouse region of eastern Washington, USA, is a large producer of dry pea (*Pisum sativum*). The main pest of dry pea in the Palouse is pea aphid (*Acyrthosiphon pisum* Harris), a specialist herbivore that transmits pathogens such as PEMV (Chisholm et al., 2018; Clark et al., 2023). PEMV is a persistently transmitted, bipartite ds‐RNA virus that attacks many legume species and causes severe damage to dry peas (Basu et al., 2021). Diverse aphid predators exist in pea fields and include foliar species like lady beetles (Coccinellidae), *Nabis* spp. (Nabidae), and big‐eyed bugs (Geocoridae), and ground‐foraging beetles (Carabidae and Staphylinidae) (Sandhi & Reddy, 2020). We focused on two foliar predators, the lady beetles *Coccinella septempunctata* and *Hippodamia convergens*, and a ground predator, the carabid *Pterostichus melanarius*. These species were chosen in part because the two lady beetles were expected to broadly overlap in their foraging niches, while both species were expected to differ in their foraging niches from *P. melanarius* (Lee et al., 2022; Losey & Denno, 1998a). Including two lady beetle species also allowed us to examine the role of foraging niches relative to other factors like voracity, disturbance, or competitive interference. In addition, these three predator species are common and co‐occur in pea agroecosystems and are the main predators of *A. pisum* (Snyder & Clevenger, 2004; Youssef, 2000). By manipulating the number and composition of predator communities, we were able to assess effects on pea aphids and the PEMV pathogen.

### Background theory and justification

**FIGURE 1:**
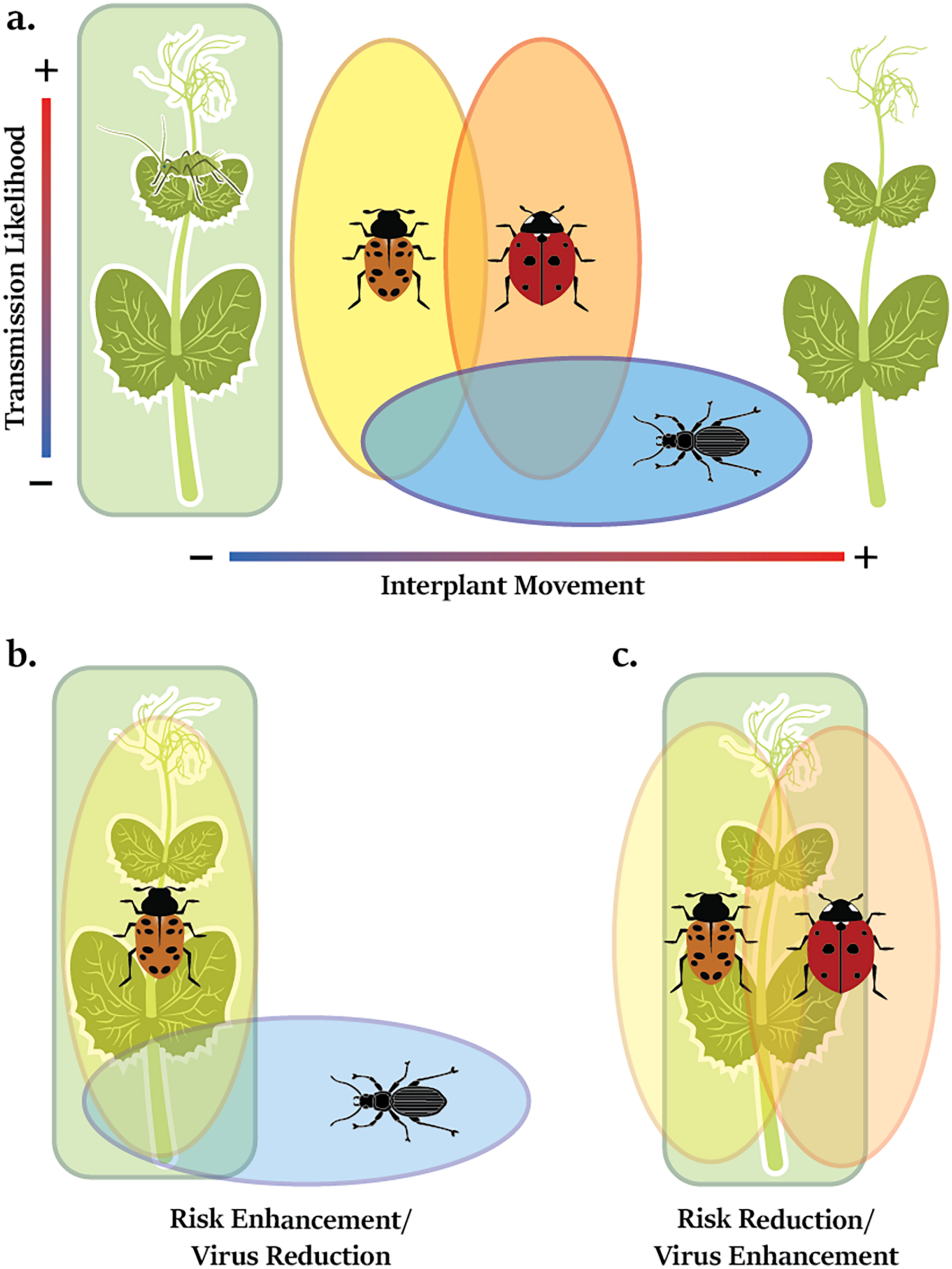
Theorized multiple predator effects on prey and virus transmission contingent on habitat domain overlap in a multi‐host system. (a) Aphid vectors' habitat domain consists of entire host plants, with their capacity to vector pathogens determined by abundance, interplant movement, and on‐host feeding location. (b) Predators with distinct habitat domains may enhance predation risk by preventing prey escape and reducing virus transmission through the capture of dispersing vectors. (c) Predators with overlapping habitat domains are predicted to reduce predation risk due to interference interactions and enhance virus transmission by stimulating greater interplant movement. Image credits: orange lady beetle (*Hippodamia convergens*), aphid, and pea plant by Megan Asche; red lady beetle (*Coccinella septempunctata*), and ground beetle (*Pterostichus melanarius*) by Benjamin W. Lee.

The habitat domain theory predicts the efficiency of predation based on the overlap of niches for each predator species in the community (Northfield et al., 2017; Preisser et al., 2007; Schmitz, 2007). Using this framework, we predicted how predators may alter vector behaviors that affect PEMV transmission (Figure 1). A pea aphid's ability to transmit pathogens is determined by a wide range of factors such as their abundance, feeding location and duration, host species preference, and rates of movement between hosts (Chisholm et al., 2019; Finke, 2012; Pinheiro et al., 2017). Predators affect aphid feeding locations and movement between hosts and may; therefore, exhibit behaviorally mediated impacts on PEMV transmission. When feeding on its preferred hosts in *Fabaceae*, pea aphid habitat often includes the entirety of the plant, overlapping fully with the niches of foliar‐foraging predators like *H. convergens* and *C. septempunctata* and partially with ground‐foraging predators like *P. melanarius* (Figure 1a) (Lee et al., 2022; Losey & Denno, 1998a). In response to predation, pea aphids also engage in avoidance behaviors specific to the nature of the threat (Lee et al., 2022; Losey & Denno, 1998a, 1998b). When attacked by foliar predators, aphids can drop from host plants where they are susceptible to predation by ground‐foraging predators like *P. melanarius* that can only access aphids on the lower portions of hosts and the ground (Lee et al., 2022; Losey & Denno, 1998a; Snyder & Ives, 2001). PEMV transmission occurs more readily when aphids feed on younger plant tissue (Chisholm et al., 2018). Thus, predators that cause aphids to move lower on plants may decrease PEMV transmission by causing aphids to feed on older plant tissue, while predator avoidance behaviors that shift aphids to younger tissue may increase the risk of PEMV transmission.

Combinations of foliar‐ and ground‐foraging predators may have either additive or super‐additive (higher than what each species could do alone) ability to consume aphids, as aphids are either unable to escape from foliar predators or leave the host and become vulnerable to ground predators (Losey & Denno, 1998a; Schmitz, 2007). We expected virus transmission to decrease as ground predators reduce the dispersal of both disturbed and naturally dispersing aphids (Figure 1b). Multiple foliar‐foraging predators with overlapping habitats are predicted to reduce predation through interference competition (Schmitz, 2007), though species' preference for different on‐plant foraging locations may result in niche complementarity and greater prey suppression (Straub & Snyder, 2008). In either scenario, aphids may avoid predation by moving between hosts, promoting plant‐to‐plant transmission (Figure 1c). Additionally, aphids may seek refuge on the tops of plants where smaller surfaces reduce predator access, accelerating transmission by feeding on more susceptible tissue (Chisholm et al., 2019) (Figure 1b,c).

### Field experiment

To test our predictions, we conducted a field experiment to evaluate multiple predator effects on: (1) aphid abundance, (2) aphid feeding behavior and movement, and (3) PEMV prevalence. Pea aphids originated from individuals collected in commercial pea fields in Washington and were maintained on potted pea plants (*P. sativum* cv. “Banner”) at Washington State University (Pullman, WA, USA), in greenhouses (16:8 h light:dark; 21–24°C during light; 16–18°C during dark). Our PEMV isolate was obtained from the University of Idaho (Moscow, ID, USA) and maintained by transferring aphids fed on PEMV‐infected peas into uninfected colonies, introducing clean plants as needed. Samples from infectious and uninfectious colonies were tested monthly for the presence of PEMV using reverse transcription‐polymerase chain reaction (RT‐PCR); these samples confirmed 100% infection levels in the infectious aphid colony and 0% in the uninfectious colony.

Adult *C. septempunctata* (C7) and *H. convergens* (HC) individuals were hand‐collected from pea and alfalfa fields and adjacent weedy foliage in Eastern Washington State. Adult *P. melanarius* (PT) were collected from the same locations using 10 × 7.5 cm plastic pitfall traps buried flush with the soil for 24 h. All predators were held for up to 2 weeks before experiments in growth chambers at 22°C in 9 × 50 mm Petri dishes and provided a moist cotton ball and ad libitum pea aphids, which were readily consumed. Predators were starved for 24 h prior to use in the experiments to encourage active foraging and predation behavior.

The field experiment was conducted in two blocks from June to July 2019 on bare‐soil plots at the Palouse Conservation Farm in Pullman, WA, USA. Pea plants (cv “Banner”) were grown in greenhouses for 2 weeks in potting soil (Sun Gro Sunshine LC1 Grower Mix) prior to use. For each replicate, a 3 × 3 grid of plants spaced 40 cm apart was buried in pots and covered with soil, then covered with a 60 × 60 × 60 cm mesh tent to prevent the escape of insects. For each replicate, twenty‐five 7‐day‐old PEMV‐infectious *A. pisum* apterous adults were placed at the base of the center pea plant and confined within a mesh barrier for 24 h to establish. After 24 h, the mesh barrier was removed and predators were added. Cages were assigned one of six predator treatments or a no‐predator control. Predator treatments were either 4 individuals from each single species, or 2 individuals of each of two species in a pair, with 3 unique single species and 3 unique pairs of species. This substitutive design allowed us to examine the effects of individual species and species pairs with constant densities. Predators and aphids foraged freely, and aphid abundance and feeding location on each plant were recorded every 2 days for 6 days. Plants were carefully examined using dental mirrors to ensure all aphids were counted and to minimize researcher disturbance of aphids. Predators found dead were replaced, though we did not observe intraguild predation, as all dead predators were intact. After 6 days, all aphids were removed with aspirators, and each pot received a granular formulation of imidacloprid (Bayer Crop Science, NJ, USA) watered into the soil to kill aphids. Plants then grew for 7 days to allow PEMV to develop.

After 7 days, aboveground tissue was collected, frozen in liquid nitrogen, and stored at −80°C. PEMV prevalence was assessed using RT‐PCR with PEMV‐CP‐specific primers (Appendix S1: Table S1) designed from conserved regions of CP from multiple isolates of PEMV (Altschul et al., 1990) and examining electrophoresis gel bands from PCR products. We completed RNA extraction using Promega SV total RNA isolation kits (Promega, Madison, WI, USA). cDNA was synthesized from 1 μg of total RNA using Bio‐Rad iScript cDNA synthesis kits, and PCR was carried out using DreamTaq Green PCR Master Mix (Thermo Fisher Scientific, Inc., Waltham, MA, USA) and PEMV primers for the coat protein (Appendix S1: Table S1). The bands were quantified using ImageJ software for relative PEMV abundance, and the amplified DNA bands were eluted from the gel using DNA Gel Extraction (ThermoFisher Scientific) using the manufacturer's protocol and sequenced with the help of Washington State University Genomic Core Lab Facility using Sanger sequencing methodology to confirm the amplification of PEMV.

### Statistical analysis

To assess the effects of predator treatments on aphids and PEMV, we fit generalized linear mixed models (GLMMs) to data. For aphid abundance, feeding location, and dispersal, models included densities of HC, C7, and PT, day (2, 4, or 6), and all two‐way interactions as fixed effects, with cage as a random effect. Models were limited to two‐way effects as we did not include three‐species treatments. We assumed aphid abundance had a negative binomial distribution to account for overdispersion in the count data. The proportion of aphids feeding on the upper half of host plants (feeding location) and the proportion of aphids dispersed from the released plant (dispersal) were examined using a binomial distribution with a “logit” link function. For PEMV prevalence, we ran two models on how aphid behavior and predator treatments affected prevalence using GLMMs with a binomial distribution including parameters describing the proportion of infected plants (*p*) and the number of host plants within each mesocosm (*n*). The first model investigated the effects of aphid responses on PEMV prevalence and included aphid abundance, feeding location, and dispersal from days 2, 4, and 6 as fixed effects, with cage as a random effect. The second model assessed the direct effects of predator treatments on PEMV prevalence and included densities of HC, C7, and PT, and all two‐way interactions as fixed effects; as predator densities were constant throughout the experiment this final model did not include repeated measures.

We next conducted statistical inference tests by systematically removing key parameters from models and evaluating model fit. For each response, we began with GLMMs previously described, which included all two‐way interactions between predator treatments. We conducted likelihood ratio tests to compare this full model to models with a single interaction term removed (i.e., HC:C7, HC:PT, or C7:PT) or all interactions removed. The interaction terms describe more aphids (positive interaction) or fewer aphids (negative interaction) than predicted by single‐species treatments (additive effect), which can indicate risk reduction or risk enhancement, respectively (Northfield et al., 2014). A log‐link function was used, assuming a multiplicative risk model (Sih et al., 1998). Thus, a statistical rejection of a simplified model indicated that the interaction or interactions removed in the “reduced model” improved model fit. All analyses were conducted using R v 3.5.2 (R Development Core Team, 2018). GLMMs were run using the “glmmTMB” package (Brooks et al., 2017), significance tests based on Wald tests from the “glmmTMB” package, and likelihood ratio tests using the “car” package (Fox & Weisberg, 2018).

## RESULTS

### Effects of predators on aphid responses and PEMV prevalence

**FIGURE 2:**
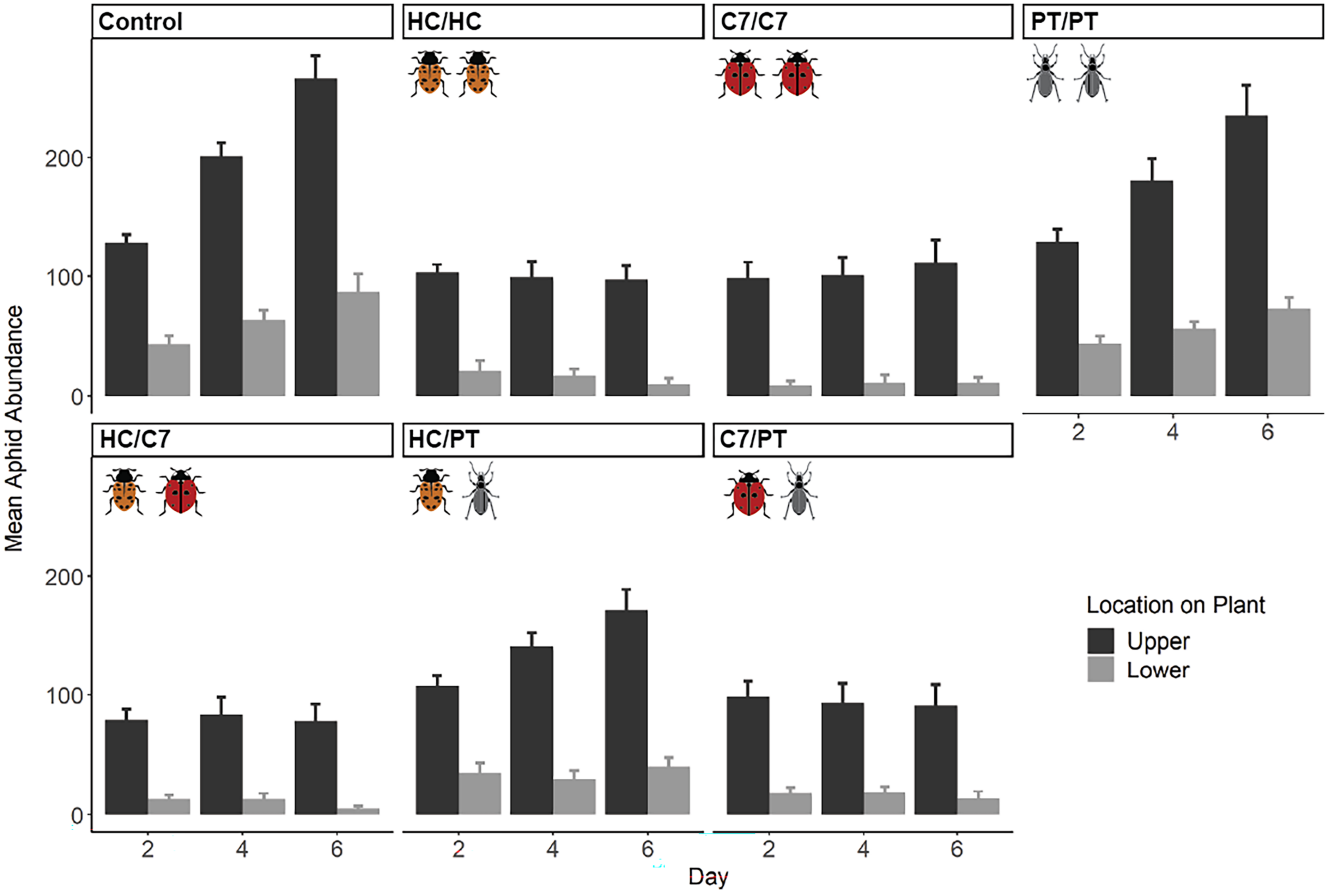
Effects of predator treatments on mean aphid abundance and feeding location over time. Bars represent values from predator treatments, while points represent values from no‐predator controls. Error bars indicate 95% CIs. Image credits: orange lady beetle (*Hippodamia convergens* [HC]) by Megan Asche; red lady beetle (*Coccinella septempunctata* [C7]), and ground beetle (*Pterostichus melanarius* [PT]) by Benjamin W. Lee.

Predators reduced aphid abundance except for the single‐species *P. melanarius* treatment (Figure 2) (HC: GLMM, *Z* = −5.53, *p* < 0.001; C7: *Z* = −5.63, *p* < 0.001; PT: *Z* = −1.45, *p* = 0.15). Combining *C. septempunctata* and *P. melanarius* reduced aphid abundance beyond that of either single species alone (*Z* = −2.88, *p* = 0.004). The presence of either lady beetle species increased the proportion of aphids feeding on the upper half of plants (Figure 2); *C. septempunctata* produced this effect throughout (*Z* = 3.87, *p* < 0.001), while the effect of *H. convergens* increased over time (*Z* = 5.70, *p* < 0.001). *C. septempunctata* induced greater aphid dispersal from its starting host to a recipient host (*Z* = 2.43, *p* = 0.015). Pairing *H. convergens* and *P. melanarius* did not significantly reduce aphid dispersal (*Z* = −1.40, *p* = 0.16).

**FIGURE 3:**
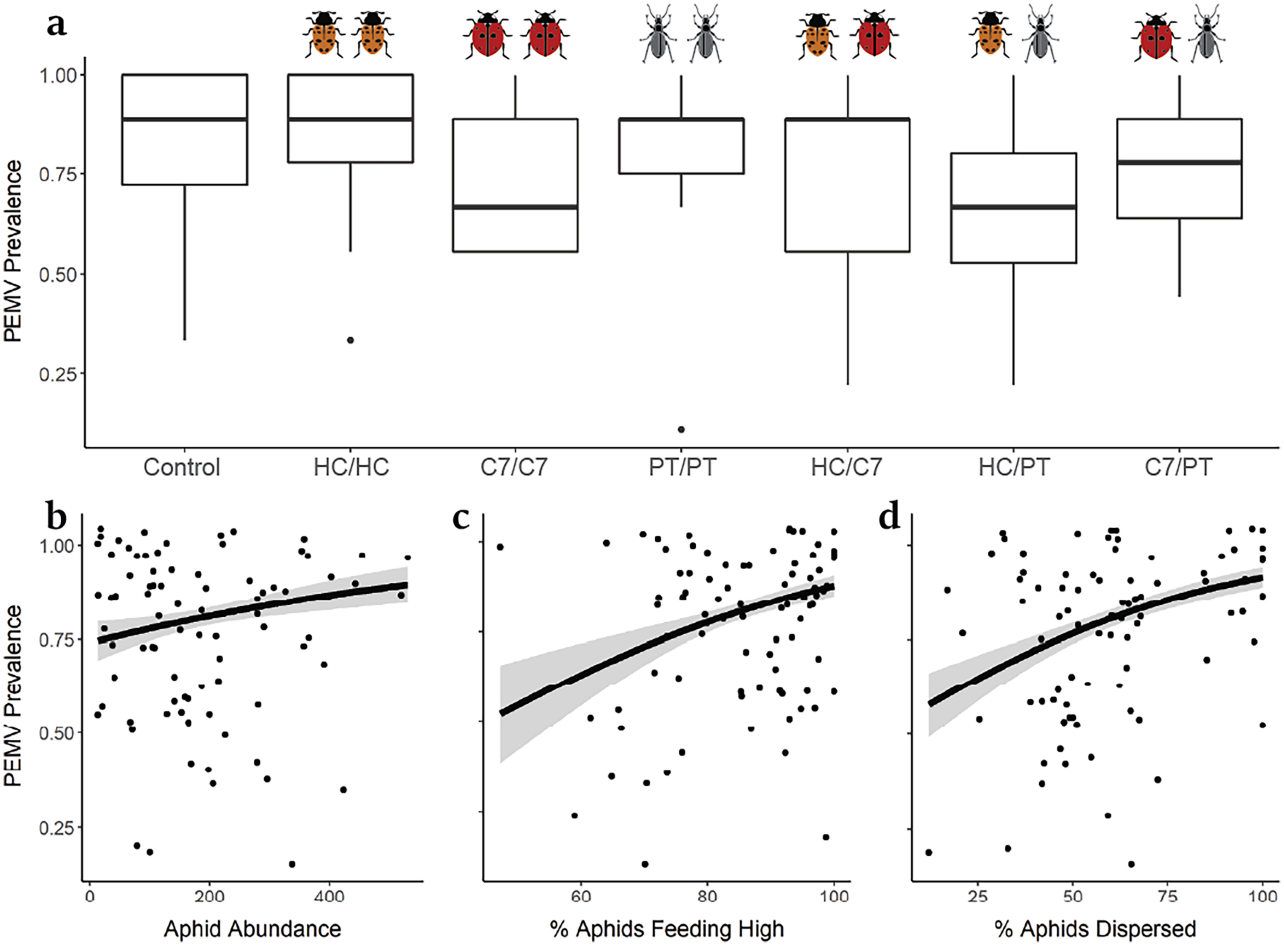
Effects of (a) paired predator treatments and (b–d) aphid abundance, feeding location, and dispersal on pea‐enation mosaic virus (PEMV) prevalence in field mesocosms. (b–d) Lines indicate predicted slope from binomial generalized linear mixed model, and β and *p*‐values represent coefficients and significance tests from the model (Appendix S1: Table S2). Image credits: orange lady beetle (*Hippodamia convergens* [HC]) by Megan Asche; red lady beetle (*Coccinella septempunctata* [C7]), and ground beetle (*Pterostichus melanarius* [PT]) by Benjamin W. Lee.

PEMV prevalence was lowest in cages with paired *H. convergens* and *P. melanarius* (χ^2^ = 3.85, *p* = 0.049), with no strong effects of other treatments (Figure 3a; Appendix S1: Table S2). Aphid abundance was not significantly correlated with PEMV prevalence (*Z* = 1.50, *p* = 0.13, Figure 3b). PEMV prevalence was greater when aphids fed higher on host plants (*Z* = 3.28, *p* = 0.012, Figure 3c) and when more aphids dispersed between hosts (*Z* = 3.28, *p* = 0.001, Figure 3d).

### Effects of predator diversity

**FIGURE 4:**
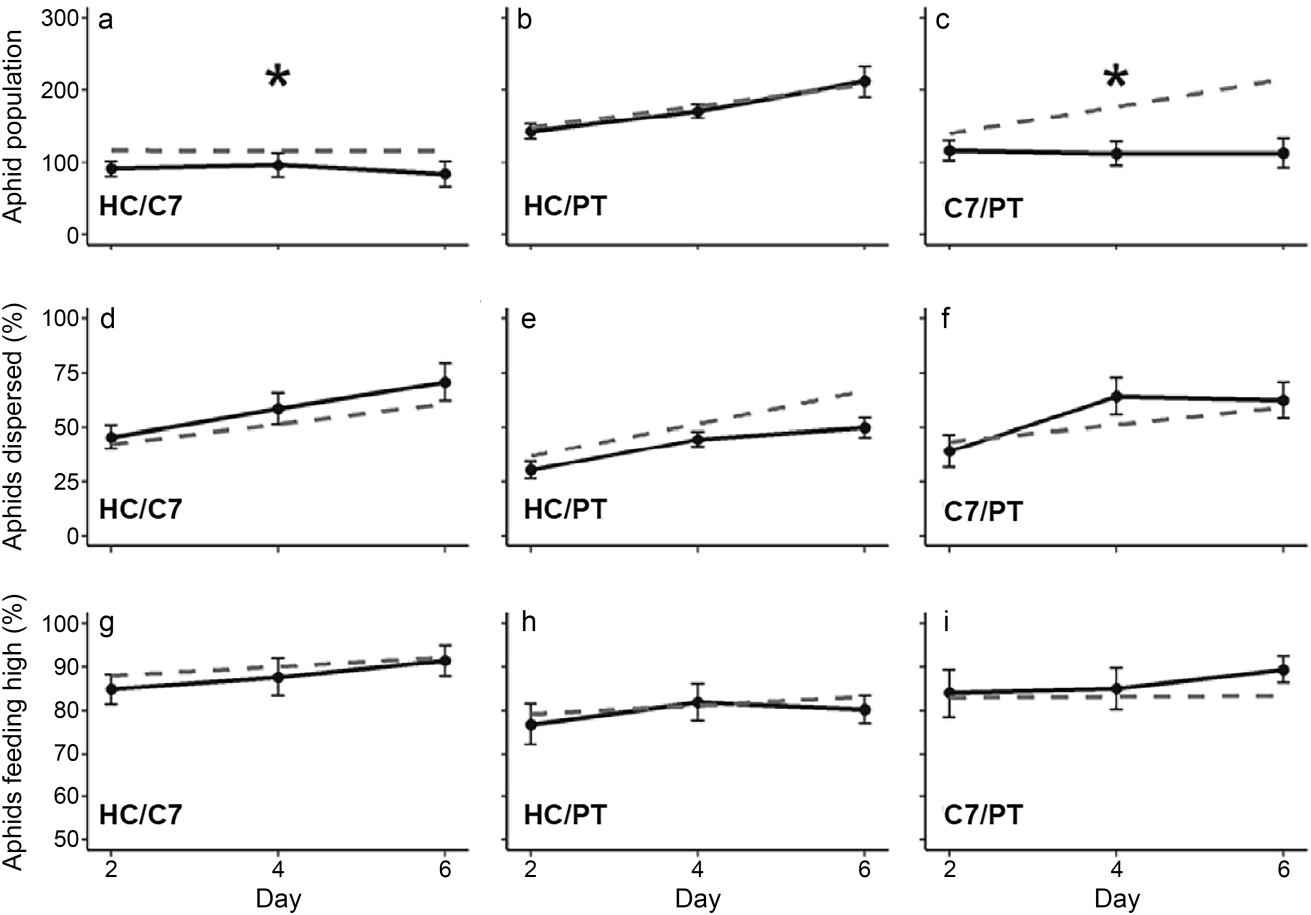
Comparisons of observed effects of multiple predator species treatments (solid black) to expected additive effects (dotted gray) on (a–c) aphid population, (d–f) dispersal, and (g–i) feeding location; species abbreviations are HC = *Hippodamia convergens*; C7 = *Coccinella septumpunctata*; PT = *Pterosticus melanarius*. Expected additive effects were calculated using mean observed values from single‐species treatments adjusted to reflect predator densities used in multiple‐species treatments. Asterisks indicate models including predator diversity interaction significantly improved fit according to likelihood ratio tests (Appendix S1: Table S3, α = 0.10). Error bars represent 90% CIs.

Removing all interaction terms as an overall diversity effect test significantly reduced model fit, showing predator diversity affected PEMV (χ^2^ = 10.49, df = 3, *p* = 0.015). When model terms were evaluated, model fit improved when models included interactions between *H. convergens* and *C. septempunctata* (χ^2^ = 3.07, df = 1, *p* = 0.079), *C. septempunctata* and *P. melanarius* (χ^2^ = 7.89, df = 1, *p* = 0.005), but not between *H. convergens* and *P. melanarius* (χ^2^ = 0.22, df = 1, *p* = 0.64). This suggests predator diversity contributed to reduced aphid abundance and greater predation risk, beyond the additive effects of each species when *C. septempunctata* was paired with *H. convergens* or *P. melanarius* (Figure 4a–c; Appendix S1: Table S3). For models of aphid feeding location and dispersal, removal of interactions did not affect model fit, indicating no sub‐ or super‐additive predator diversity effects (Figure 4d–i; Appendix S1: Table S3).

## DISCUSSION

Disease ecology models that consider community‐wide interactions suggest that predator‐induced changes in vector behavior can contribute more to virus transmission than vector abundance (Crowder et al., 2019). Our study supports this view by showing that multiple predator species enhanced predation, whether predators occupy distinct or overlapping habitat domains, but the effects of multiple predator species on feeding location contributed more to PEMV prevalence than effects on aphid abundance. This suggests limitations of using predator habitat domain theory to predict emergent effects of diversity on virus transmission and that the framework should be expanded to capture prey behavior. Projecting effects of increasing predator diversity on pathogen transmission and ecosystem functions thus requires careful evaluation of prey abundance and behavior, and specific predator–predator and predator–prey interactions.

Both *H. convergens* and *C. septempunctata* reduced aphid populations (Figure 2), and while we predicted interference may reduce predation, the two lady beetles paired enhanced predation beyond additive effects (Figure 4). Studies show that intraspecific competition may exert greater effects than interspecific competition in lady beetles when species partition plant space (Straub & Snyder, 2008). Our study also used adult lady beetles that rarely engage in intraguild predation (Rondoni et al., 2012). Predation enhancement was strongest with *C. septempunctata* and *P. melanarius*, supporting niche complementarity between them. Yet, predation enhancement was not observed with *H. convergens* and *P. melanarius* (Figure 4b). *C. septempunctata* is larger than *H. convergens* and may more readily induce aphids to drop from hosts and become vulnerable to ground beetles (Hoki et al., 2014). Despite strong effects on aphid abundance, lady beetles did not reduce aphid populations below ~100 individuals, and the majority fed on the upper half of plants where PEMV transmission is more likely (Chisholm et al., 2019). Because there was no combination of predators that lowered aphid populations enough to impact disease prevalence, our study suggests that natural enemies alone may not be sufficient for aphid and PEMV control, though longer timeframes and other external factors could significantly affect predation outcomes. Foliar predators often have difficulty accessing prey at the top of plants, which serve as refuges for aphids (Grevstad & Klepetka, 1992). Given that feeding location was driven by predator identity (Figure 4g–i; Appendix S1: Table S3), increasing predator biodiversity may affect feeding location and transmission if additional species access prey in refuges (Northfield et al., 2012). The existence and location of refuges on plants and the relative susceptibility of host structures may mediate how multiple predator effects on vector feeding location determine transmission outcomes. Further studies examining additional predator species with varied abundances, behaviors, phenologies, and prey preferences could identify other avenues through which predators can impact transmission.

Increased rates of vector dispersal could accelerate virus transmission if more susceptible hosts are encountered, or reduce transmission if feeding is interrupted (Crowder et al., 2019). We show aphid dispersal promoted PEMV prevalence (Figure 3d), suggesting greater contact with additional hosts promoted transmission. We predicted pairs of foliar‐ and ground‐foraging predators may reduce vector dispersal by increasing predation risk associated with movement, while foliar predators with overlapping habitats would increase aphid dispersal by inducing aphids to drop from hosts (Figure 1). Yet, predators with fully overlapping habitat domains (HC and C7) did not increase aphid dispersal within mesocosms (Figure 4d–f). It is possible that enhanced predation by *H. convergens* and *C. septempunctata* masked effects on aphid movement if dispersing aphids were more easily captured. In support of predictions, mesocosms with *H. convergens* and *P. melanarius* had the lowest rates of aphid dispersal and PEMV prevalence (Figure 3a; Appendix S1: Table S2). Interestingly, *H. convergens* and *P. melanarius* were the only diversity treatments where there was no synergistic effect on predation rates. An intermediate intensity of predation, like that posed by a low density of *H. convergens*, may reduce abundance without generating colony‐wide disturbance, limiting behaviors associated with transmission.

Consistently, the presence of *C. septempunctata* increased aphid dispersal, heightened feeding location, and enhanced other predators' ability to suppress aphid abundance (Appendix S1: Table S2). *C. septempunctata* is a non‐native species in North America with increased voracity, perhaps due to larger body size, and demonstrated competitive advantages over native species (Lee et al., 2018; Petersen & Losey, 2024). The outsized role of a single predator species indicates that species identity can drive how predators indirectly affect transmission. Highly disruptive predator species may be more likely to generate prey avoidance behaviors that allow for synergistic multiple predator effects to manifest, while predators that consume aphids with minimal disturbance may function additively (Losey & Denno, 1998b). Though the predator habitat domain will likely determine how prey react to disturbance (i.e., seeking refuge) (Schmitz et al., 2017), the frequency or intensity of predator disturbance may influence the strength of interactive effects between predators.

This initial examination of how spatial theories of multiple predator effects might predict pathogen transmission highlights the importance of prey behavior and documents how specific characteristics of systems can mediate diversity effects. In general, enhanced predation risk resulting from increased predator diversity did not reduce virus prevalence, as predators induced aphid vector behaviors that increased transmission, though this effect may be diminished at longer time scales as population effects compound. Given that behavioral responses were driven more by predator and host characteristics (i.e., presence of refuges) than by predator–predator interactions, habitat domain theory may not fully capture vector responses and pathosystem characteristics important to local transmission. By affecting the habitat available for predators to exploit, host plant structure may be an important determinant of how predators affect pathogen transmission if vectors within refuges can escape predation. Increasing predator diversity could thus reduce transmission if species better suited to accessing refuges, such as parasitoid wasps, are present (Northfield et al., 2012), even if effects on total prey abundance are similar. Space use within habitats, where species may reduce interference by partitioning subsections of hosts, may also complicate assumptions about the nature of predator–predator interactions.

While our study did not fully support predictions for multiple predators' effects on vector abundance and behavior, habitat domain theory serves as a useful tool to guide experimentation on species interactions. Recent efforts to expand the habitat domain framework have focused on explaining patterns of predator–prey interactions across landscapes (Schmitz et al., 2017). Indeed, across larger spatial and temporal scales, the relative contributions of vector abundance and behaviors to pathogen spread will vary as vectors make foraging and movement decisions in response to changes in population density, host conditions, and levels of predation risk (Crowder et al., 2019; Culshaw‐Maurer et al., 2020). Though our study was limited in duration and examined only a specific pathosystem, given the significance of individual vector behaviors to rates of transmission, experiments examining specific predator–predator and predator–prey interactions over longer periods are required to detect emergent effects and improve predictions.

## AUTHOR CONTRIBUTIONS

*Conceptualization*: Benjamin W. Lee and David W. Crowder. *Methodology*: Benjamin W. Lee, Tobin D. Northfield, and Saumik Basu. *Writing—review and editing*: Benjamin W. Lee, Liesl Oeller, Tobin D. Northfield, and David W. Crowder. *Funding acquisition*: Benjamin W. Lee and David W. Crowder. All authors have read and agreed to the published version of the manuscript.

## CONFLICT OF INTEREST STATEMENT

The authors declare no conflicts of interest.

## Supporting information


Appendix S1.

## Data Availability

Data (Lee et al., 2025) are available in Zenodo at https://doi.org/10.5281/zenodo.15320017.

## References

[eap70065-bib-0001] Altschul, S. F. , W. Gish , W. Miller , E. W. Myers , and D. J. Lipman . 1990. “Basic Local Alignment Search Tool.” Journal of Molecular Biology 215: 403–410. 2231712 10.1016/S0022-2836(05)80360-2

[eap70065-bib-0002] Basu, S. , R. E. Clark , S. Bera , C. L. Casteel , and D. W. Crowder . 2021. “Responses of Pea Plants to Multiple Antagonists Are Mediated by Order of Attack and Phytohormone Crosstalk.” Molecular Ecology 30: 4939–4948. 34347913 10.1111/mec.16103

[eap70065-bib-0003] Brooks, M. E. , K. Kristensen , K. J. van Benthem , A. Magnusson , C. W. Berg , A. Nielsen , H. J. Skaug , M. Maechler , and B. M. Bolker . 2017. “glmmTMB Balances Speed and Flexibility among Packages for Zero‐Inflated Generalized Linear Mixed Modeling.” The R Journal 9: 378–400.

[eap70065-bib-0004] Chisholm, P. J. , S. D. Eigenbrode , R. E. Clark , S. Basu , and D. W. Crowder . 2019. “Plant‐Mediated Interactions between a Vector and a Non‐Vector Herbivore Promote the Spread of a Plant Virus.” Proceedings of the Royal Society B: Biological Sciences 286: 20191383. 10.1098/rspb.2019.1383 PMC6784723 31551062

[eap70065-bib-0005] Chisholm, P. J. , N. Sertsuvalkul , C. L. Casteel , and D. W. Crowder . 2018. “Reciprocal Plant‐Mediated Interactions between a Virus and a Non‐Vector Herbivore.” Ecology 99: 2139–2144. 29999522 10.1002/ecy.2449

[eap70065-bib-0006] Clark, R. E. , S. Basu , S. D. Eigenbrode , L. C. Oeller , and D. W. Crowder . 2023. “Risk Assessment for Non‐Crop Hosts of Pea Enation Mosaic Virus and the Aphid Vector *Acyrthosiphon pisum* .” Agricultural and Forest Entomology 3: 427–434.

[eap70065-bib-0007] Crowder, D. W. , J. Li , E. T. Borer , D. L. Finke , R. Sharon , D. E. Pattemore , and J. Medlock . 2019. “Species Interactions Affect the Spread of Vector‐Borne Plant Pathogens Independent of Transmission Mode.” Ecology 100: 1–10. 10.1002/ecy.2782 31170312

[eap70065-bib-0008] Culshaw‐Maurer, M. , A. Sih , and J. Rosenheim . 2020. “Bugs Scaring Bugs: Enemy‐Risk Effects in Biological Control Systems.” Ecology Letters 23: 1693–1714. 32902103 10.1111/ele.13601 PMC7692946

[eap70065-bib-0009] Eigenbrode, S. D. , N. Bosque‐Pérez , and T. S. Davis . 2018. “Insect‐Borne Plant Pathogens and Their Vectors: Ecology, Evolution, and Complex Interactions.” Annual Review of Entomology 63: 169–191. 10.1146/annurev-ento-020117-043119 28968147

[eap70065-bib-0010] Fereres, A. , and A. Moreno . 2009. “Behavioural Aspects Influencing Plant Virus Transmission by Homopteran Insects.” Virus Research 141: 158–168. 19152819 10.1016/j.virusres.2008.10.020

[eap70065-bib-0011] Finke, D. L. 2012. “Contrasting the Consumptive and Non‐Consumptive Cascading Effects of Natural Enemies on Vector‐Borne Pathogens.” Entomologia Experimentalis et Applicata 144: 45–55.

[eap70065-bib-0012] Fox, J. , and S. Weisberg . 2018. “Visualizing Fit and Lack of Fit in Complex Regression Models with Predictor Effect Plots and Partial Residuals.” Journal of Statistical Software 87: 1–27.

[eap70065-bib-0013] Grevstad, F. S. , and B. W. Klepetka . 1992. “The Influence of Plant Architecture on the Foraging Efficiencies of a Suite of Ladybird Beetles Feeding on Aphids.” Oecologia 92: 399–404. 28312606 10.1007/BF00317466

[eap70065-bib-0014] Hoki, E. , J. Losey , and T. A. Ugine . 2014. “Comparing the Consumptive and Non‐Consumptive Effects of a Native and Introduced Lady Beetle on Pea Aphids (*Acyrthosiphon pisum*).” Biological Control 70: 78–84.

[eap70065-bib-0015] Ives, A. R. , B. J. Cardinale , and W. E. Snyder . 2005. “A Synthesis of Subdisciplines: Predator‐Prey Interactions, and Biodiversity and Ecosystem Functioning.” Ecology Letters 8: 102–116.

[eap70065-bib-0016] Jones, R. A. C. , and R. A. Naidu . 2019. “Global Dimensions of Plant Virus Diseases: Current Status and Future Perspectives.” Annual Review of Virology 6: 387–409. 10.1146/annurev-virology-092818-015606 31283443

[eap70065-bib-0017] Lee, B. W. , S. Basu , L. Oeller , T. Northfield , and D. Crowder . 2025. “Data and Code from: Predator Niche Overlap Predicts Effects on Aphid Vectors and A Vector‐Borne Virus.” Zenodo. 10.5281/zenodo.15320017.40605552

[eap70065-bib-0018] Lee, B. W. , S. Basu , S. Bera , C. L. Casteel , and D. W. Crowder . 2021. “Responses to Predation Risk Cues and Alarm Pheromones Affect Plant Virus Transmission by an Aphid Vector.” Oecologia 196: 1005–1015. 34264386 10.1007/s00442-021-04989-6

[eap70065-bib-0019] Lee, B. W. , R. E. Clark , S. Basu , and D. W. Crowder . 2022. “Predators Affect a Plant Virus through Density and Trait‐Mediated Indirect Effects on Vectors.” Food Webs 33: e00251.

[eap70065-bib-0020] Lee, B. W. , T. A. Ugine , and J. E. Losey . 2018. “An Assessment of the Physiological Costs of Autogenous Defenses in Native and Introduced Lady Beetles.” Environmental Entomology 47: 1030–1038. 29846514 10.1093/ee/nvy068

[eap70065-bib-0021] Long, E. Y. , and D. L. Finke . 2015. “Predators Indirectly Reduce the Prevalence of an Insect‐Vectored Plant Pathogen Independent of Predator Diversity.” Oecologia 177: 1067–1074. 25561170 10.1007/s00442-014-3194-1

[eap70065-bib-0022] Losey, J. E. , and R. F. Denno . 1998a. “Positive Predator‐Predator Interactions: Enhanced Predation Rates and Synergistic Suppression of Aphid Populations.” Ecology 79: 2143–2152.

[eap70065-bib-0023] Losey, J. E. , and R. F. Denno . 1998b. “The Escape Response of Pea Aphids to Foliar‐Foraging Predators: Factors Affecting Dropping Behaviour.” Ecological Entomology 1: 53–61.

[eap70065-bib-0024] Nelson, E. H. , C. E. Matthews , and J. A. Rosenheim . 2004. “Predators Reduce Prey Population Growth by Inducing Changes in Prey Behavior.” Ecology 85: 1853–1858.

[eap70065-bib-0025] Northfield, T. D. , B. T. Barton , and O. J. Schmitz . 2017. “A Spatial Theory for Emergent Multiple Predator‐Prey Interactions in Food Webs.” Ecology and Evolution 7: 6935–6948. 28904773 10.1002/ece3.3250 PMC5587500

[eap70065-bib-0026] Northfield, T. D. , D. W. Crowder , T. Takizawa , and W. E. Snyder . 2014. “Pairwise Interactions between Functional Groups Improve Biological Control.” Biological Control 78: 49–54.

[eap70065-bib-0027] Northfield, T. D. , G. B. Snyder , A. R. Ives , and W. E. Snyder . 2010. “Niche Saturation Reveals Resource Partitioning among Consumers.” Ecology Letters 13: 338–348. 20455919 10.1111/j.1461-0248.2009.01428.x

[eap70065-bib-0028] Northfield, T. D. , W. E. Snyder , G. B. Snyder , and S. D. Eigenbrode . 2012. “A Simple Plant Mutation Abets a Predator‐Diversity Cascade.” Ecology 93: 411–420. 22624322 10.1890/11-0399.1

[eap70065-bib-0029] Petersen, M. J. , and J. E. Losey . 2024. “Niche Overlap with an Exotic Competitor Mediates the Abundant Niche‐Centre Relationship for a Native Lady Beetle.” Diversity and Distributions 30: e13825.

[eap70065-bib-0030] Pinheiro, P. V. , M. Ghanim , M. Alexander , A. R. Rebelo , R. S. Santos , B. C. Orsburn , S. Gray , and M. Cilia . 2017. “Host Plants Indirectly Influence Plant Virus Transmission by Altering Gut Cysteine Protease Activity of Aphid Vectors.” Molecular & Cellular Proteomics 16: S230–S243. 27932519 10.1074/mcp.M116.063495 PMC5393385

[eap70065-bib-0031] Preisser, L. , L. Orrock , and J. Schmitz . 2007. “Predator Hunting Mode and Habitat Domain Alter Nonconsumptive Effects in Predator‐Prey.” Ecology 88: 2744–2751. 18051642 10.1890/07-0260.1

[eap70065-bib-0032] R Development Core Team . 2018. R Version 3.5.2. R: A Language and Environment for Statistical Computing. Vienna: R Foundation for Statistical Computing.

[eap70065-bib-0033] Rondoni, G. , A. Onofri , and C. Ricci . 2012. “Laboratory Studies on Intraguild Predation and Cannibalism among Coccinellid Larvae (Coleoptera: Coccinellidae).” European Journal of Entomology 109: 353–362.

[eap70065-bib-0034] Sandhi, R. K. , and G. V. P. Reddy . 2020. “Biology, Ecology, and Management Strategies for Pea Aphid (Hemiptera: Aphididae) in Pulse Crops.” Journal of Integrated Pest Management 11: 1–20.

[eap70065-bib-0035] Schmitz, O. J. 2007. “Predator Diversity and Trophic Interactions.” Ecology 88: 2415–2426. 18027743 10.1890/06-0937.1

[eap70065-bib-0036] Schmitz, O. J. , J. R. B. Miller , A. M. Trainor , and B. Abrahms . 2017. “Toward a Community Ecology of Landscapes: Predicting Multiple Predator‐Prey Interactions across Geographic Space.” Ecology 98: 2281–2292. 28585719 10.1002/ecy.1916

[eap70065-bib-0037] Sih, A. , G. Englund , and D. Wooster . 1998. “Emergent Impacts of Multiple Predators on Prey.” Trends in Ecology & Evolution 13: 350–355. 21238339 10.1016/s0169-5347(98)01437-2

[eap70065-bib-0038] Snyder, W. E. , and G. M. Clevenger . 2004. “Negative Dietary Effects of Colorado Potato Beetle Eggs for the Larvae of Native and Introduced Ladybird Beetles.” Biological Control 3: 353–361.

[eap70065-bib-0039] Snyder, W. E. , and A. R. Ives . 2001. “Generalist Predators Disrupt Biological Control by a Specialist Parasitoid.” Ecology 82: 705–716.

[eap70065-bib-0040] Straub, C. S. , and W. E. Snyder . 2008. “Increasing Enemy Biodiversity Strengthens Herbivore Suppression on Two Plant Species.” Ecology 89: 1605–1615. 18589525 10.1890/07-0657.1

[eap70065-bib-0041] Woodcock, B. , and M. Heard . 2011. “Disentangling the Effects of Predator Hunting Mode and Habitat Domain on the Top‐Down Control of Insect Herbivores.” Journal of Animal Ecology 80: 495–503. 21155773 10.1111/j.1365-2656.2010.01790.x

[eap70065-bib-0042] Youssef, N. 2000. “The Occurrence of Coccinellids and Aphids Inhabiting Spring Wheat, Lentils, and Canola in Northern Idaho.” MS thesis, University of Idaho.

