# Supplementary material for: Predator niche overlap predicts effects on aphid vectors and a vector‐borne virus
Source: Ecol Appl. 2025 Jul 3;35(5):e70065. doi: 10.1002/eap.70065 (PMC12223468; doi:10.1002/eap.70065)
Supplement: Supplementary file 1 — Appendix S1. [file EAP-35-e70065-s001.pdf]

Predator niche overlap predicts effects on aphid vectors and a vector-borne virus

Appendix S1

Benjamin W. Lee, Saumik Basu, Liesl Oeller, Tobin D. Northfield, and David W. Crowder

Ecological Applications

Table S1. Primer pair used in this study

Gene	Primer Sequences (5′-3′)	Amplicon size (bp)
PEMP CP Fp	GTGGTGGCACCCTCTATG	290
PEMP CP Rp	GTGTCCACATGGTAGGCTATG	290

Table S2. Results of generalized linear mixed effects models on aphid abundance, feeding location, dispersal from host, and PEMV prevalence. In all models, experimental dorm was included as a random effect. Significant P values (α = .05) are shown in bold.

Response	Predictor	Estimate	SE	Z-value	P value
Aphid Abundance (Neg. Bin. Regression)	(Intercept)	4.826	0.148	32.523	0.000
	HC	-0.026	0.108	-0.239	0.811
	C7	-0.051	0.112	-0.460	0.646
	PT	0.028	0.103	0.271	0.787
	Day	0.173	0.021	8.220	0.000
	HC:C7	-0.277	0.157	-1.764	0.078
	HC:PT	0.067	0.144	0.465	0.642
	HC:Day	-0.090	0.016	-5.525	**0.000**
	C7:PT	-0.431	0.150	-2.876	**0.004**
	C7:Day	-0.098	0.017	-5.629	**0.000**
	PT:Day	-0.021	0.014	-1.452	0.147
Aphid Feeding Location (Binomial Regression)	(Intercept)	1.158	0.232	4.988	0.000
	HC	0.181	0.168	1.079	0.280
	C7	0.660	0.179	3.687	**0.000**
	PT	-0.007	0.164	-0.041	0.968
	Day	0.011	0.015	0.725	0.468
	HC:C7	-0.214	0.296	-0.723	0.470
	HC:PT	-0.155	0.274	-0.567	0.571
	HC:Day	0.079	0.014	5.703	**0.000**
	C7:PT	0.070	0.284	0.247	0.805
	C7:Day	0.026	0.016	1.648	0.099
	PT:Day	-0.009	0.011	-0.803	0.422
Aphid Dispersal (Binomial Regression)	(Intercept)	-1.246	0.233	-5.351	0.000
	HC	0.023	0.165	0.137	0.891
	C7	0.415	0.171	2.425	**0.015**
	PT	0.126	0.164	0.771	0.441
	Day	0.271	0.014	19.414	0.000
	HC:C7	0.238	0.290	0.821	0.412
	HC:PT	-0.387	0.276	-1.403	0.161
	HC:Day	0.008	0.011	0.735	0.463
	C7:PT	0.012	0.281	0.042	0.967
	C7:Day	-0.070	0.011	-6.134	**0.000**
	PT:Day	-0.001	0.010	-0.132	0.895
PEMV Prevalence (Binomial Regression)	(Intercept)	1.734	0.424	4.093	0.000
	HC	0.100	0.295	0.339	0.735
	C7	-0.260	0.290	-0.898	0.369
	PT	-0.039	0.292	-0.135	0.893
	HC:C7	-0.291	0.503	-0.578	0.563
	HC:PT	-0.941	0.479	-1.963	**0.049**

	C7:PT	-0.091	0.484	-0.189	0.850
PEMV Prevalence (Binomial Regression)	(Intercept)	-3.373	1.483	-2.274	0.023
	Aphid Abundance	0.002	0.001	1.499	0.134
	Aphid Dispersal	2.333	0.712	3.278	**0.001**
	Aphid Feeding Location	3.552	1.419	2.503	**0.012**

Table S3. Log-Likelihood (LL) values and likelihood ratio tests evaluating multiple predator effects on models predicting aphid population, dispersal, and on-host feeding location. Comparisons were between model including all two-way predator treatment interactions and models with all or individual predator treatment interactions removed. Bold P-values indicate significant contribution of interaction to model fit.

Response	Interaction Removed	LL	Chisq	DF	P- value
Aphid Abundance	None (Full Model)	-1334.6			
	All Interactions	-1339.9	10.487	3	**0.01485**
	HC*C7	-1336.2	3.071	1	**0.07969**
	HC*PT	-1334.8	0.2157	1	0.6423
	C7*PT	-1338.6	7.8994	1	**0.00495**
Aphid Dispersal	None (Full Model)	-1581.0			
	All Interactions	-1582.5	3.0312	3	0.3868
	HC*C7	-1581.3	0.6701	1	0.413
	HC*PT	-1581.9	1.9407	1	0.1636
	C7*PT	-1581.0	0.0018	1	0.9666
Aphid Feeding Location	None (Full Model)	-1235.7			
	All Interactions	-1236.2	0.9321	3	0.8177
	HC*C7	-1236.0	0.5212	1	0.4703
	HC*PT	-1235.9	0.3208	1	0.5711
	C7*PT	-1235.8	0.9605	1	0.8057
